# Result of randomized control trial to increase breast health awareness among young females in Malaysia

**DOI:** 10.1186/s12889-016-3414-1

**Published:** 2016-08-08

**Authors:** Mehrnoosh Akhtari-Zavare, Muhamad Hanafiah Juni, Salmiah Md Said, Irmi Zarina Ismail, Latiffah A. Latiff, Sima Ataollahi Eshkoor

**Affiliations:** 1 Cancer Resource & Education Center, Universiti Putra Malaysia, 43400 Serdang, Selangor Malaysia; 2 Department of Community Health, Faculty of Medicine & Health Science, Universiti Putra Malaysia, 43400 Serdang, Selangor Malaysia; 3 Department of Family Medicine, Faculty of Medicine & Health Science, Universiti Putra Malaysia, 43400 Serdang, Selangor Malaysia; 4 Malaysian Research Institute on Ageing (MyAgeing), Universiti Putra Malaysia, 43400 Serdang, Selangor Malaysia

**Keywords:** Breast cancer, Breast self-examination, Health education, Belief, Malaysia

## Abstract

**Background:**

Breast cancer is the most common cancer and the second principal cause of cancer deaths in women worldwide as well as in Malaysia. Breast self-examination (BSE) has a role in raising breast cancer awareness among women and educational programs play an important role in breast cancer preventive behavior. The aim of this study is to develop, implement and evaluate the effectiveness of Breast Health Awareness program based on health belief model on knowledge of breast cancer and breast-selfexamination and BSE practice among female students in Malaysia.

**Methods:**

A single-blind randomized controlled trial was carried out among 370 female undergraduate students from January 2011 to April 2012 in two selected public universities in Malaysia. Participants were randomized to either the intervention group or the control group. The educational program was delivered to the intervention group. The outcome measures were assessed at baseline, 6, and 12 months after implementing the health educational program. Chi-square, independent samples t-test and two-way repeated measures ANOVA (GLM) were conducted in the course of the data analyses.

**Results:**

Mean scores of knowledge on breast cancer (*p*<0.003), knowledge on breast self examination (*p*<0.001), benefits of BSE (*p*<0.00), barrier of BSE (0.01) and confidence of BSE practice (*p*<0.00) in the intervention group had significant differences in comparison with those of the control group 6 and 12 months after the intervention. Also, among those who never practiced BSE at baseline, frequency of BSE practice increased 6 and 12 months after the intervention (*p*<0.05).

**Conclusion:**

The Breast Health Awareness program based on health the belief model had a positive effect on knowledge of breast cancer and breast self-examination and practice of BSE among females in Malaysia.

**Trial registration:**

The ANZCTR clinical trial registry (ACTRN12616000831482), retrospectively registered on Jun 23, 2016 in ANZCTR.org.au.

## Background

Despite extensive progress and effort in treatment, breast cancer remains one of the most life- threatening conditions among women worldwide [1]. Similarly, in Malaysia, breast cancer is the most common type of cancer among females regardless of their ethnic groups from the age of 15 years onwards [2]. The National Cancer Registry (2008) reported that there are 11,952 registered female breast cancer cases, accounting for 31.3 % of all cancer cases registered [2].

A significant number of Malaysian women present with advanced stages of the breast cancer due to limited knowledge of breast cancer, breast cancer screening, and breast self-examination, which is considered as the main barrier among Malaysian women [3–5]. According to the Third National Health Morbidity Survey [6], the prevalence rate for breast examination in Malaysia was 70.35 %, where the highest was for breast self-examination (57.14 %), followed by clinical breast examination (51.77 %) and mammography (7.57 %) [6]. Based on the results of another studies, low percentages of clinical breast examination (23.3 %), breast self-examination (19.6 %), and intention to perform breast self-examination (18.5 %) were observed among Malaysian women [7–9]. Such results clearly show the need for awareness campaigns that raise the knowledge of young women about breast cancer and the need for the involvement of social media in promoting public breast health [6–9].

Early detection of breast cancer can reduce the mortality rate and is also important for its effective treatment [10]. Reportedly at an early stage (stage I-II), its 5-year survival rate reached 100–93 %, whereas its later detection (stages III–IV) decreased the survival rate to 72–22 % [11]. Mammography, clinical breast examination, and breast self-examination are tools for early detection of breast cancer [10]. There are arguments surrounding the efficacy of BSE. Based on the large randomized trials in Shanghai, breast self-examination was not an effective screening tool for breast cancer [12]. Likewise, the US Preventive Service Task Force and the Canadian Task Force on Preventive Health Care reported that breast self-examinations are no more beneficial for women [5, 13].

Although BSE alone is not sufficient for early detection of breast cancer, it is an effective tool for raising breast cancer awareness and the opportunity to educate women about breast cancer in developing countries [14, 15]. Moreover, the BSE training and adherence is a gateway to health promotion behavior that gives women knowledge and sets for adherence to clinical breast examination (CBE) and mammography screening guideline later in life [16, 17].

Many interventions have been done to increase breast cancer screening and BSE practice among women worldwide. For instance, Beydağ et al. [18] evaluated the effect of BSE brochures among 103 Turkish female college students at Halic University and showed that the BSE knowledge score was 43.2 ± 10.6 before education, which increased to 68.4 ± 10.5 after education (p < 0.05). Also, they found that more than half (53.4 %) of the female students, who did not perform BSE, did not have any knowledge about BSE. However, there is no randomized controlled trial done to increase the awareness of breast cancer and BSE practice among young females in Malaysia. Young women believe that they are not at risk of getting breast cancer [19]; however, the higher stages breast cancer presented among young women were more aggressive than those of older women [20].

To increase breast cancer awareness among women and build their confidence in the BSE practice, we must understand what women may or may not know about breast cancer. Also, we must understand how they feel about breast cancer and its early detection, as well as the benefits and barriers of BSE practice and other screening methods [21]. One of the most widely used conceptual frameworks, which is often used as an educational program, is the Health Belief Model (HBM) [22, 23].

**Fig. 1:**
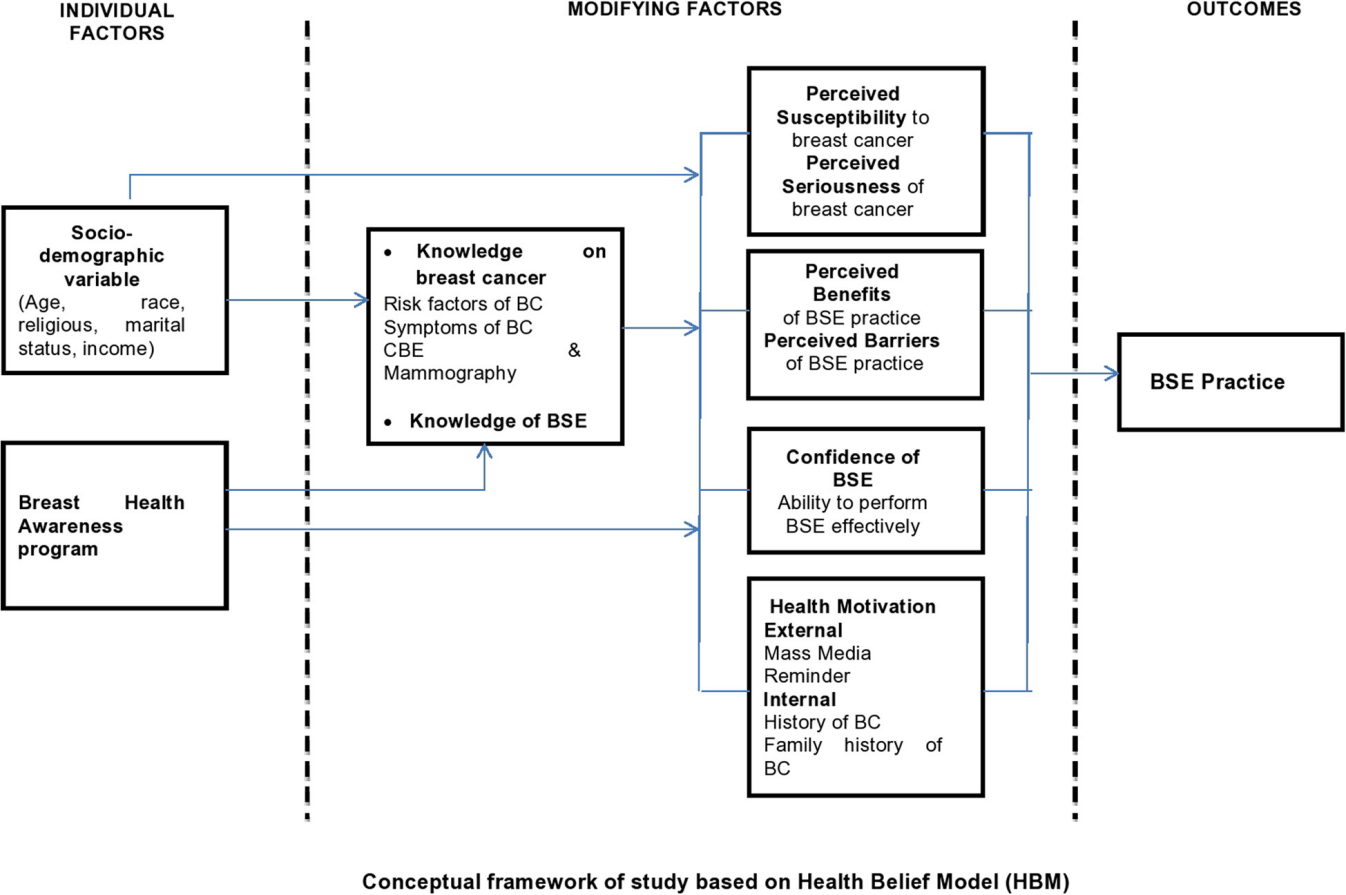
Conceptual framework of study based on Health Belief Model (HBM)

Health belief plays an important role in an individual’s interest in the health protection behaviour, which leads to screening practices in different countries and cultures [21, 24]. In this theoretical framework, women’s breast cancer screening practices such as BSE, clinical breast examination, and mammography are influenced by their health belief model [25]. This model emphasizes that health behaviour is affected by threats from health problems; for example, women perceiving susceptibility to breast cancer risk or believing that breast cancer is a serious disease are more likely to do the BSE practice. Women with higher health motivation, who perceive greater benefits and feel fewer barriers to breast examination, are more likely to perform BSE [26]. The model also suggests that, in addition to the health beliefs, the knowledge of BSE and sociodemographic background are positively related to the increase in the chances of performing BSE [27]. Figure 1 shows the components of health belief model and how it is implemented in this study.

### Purpose

The aim of this study was to determine the effectiveness of a Breast Health Awareness program based on the Health Belief Model (HBM) among female undergraduate students in public universities in Malaysia. We assumed that female students who participated in the Breast Health Awareness program would demonstrate significant differences in knowledge and beliefs towards breast cancer and breast self-examination practice compared to students who did not participate in the program.

## Methods

### Study design

A single-blinded randomized controlled trial was carried out from January 2011 until April 2012 in two selected public universities (Universiti Putra Malaysia and Universiti Teknologi MARA) in the Klang Valley in Malaysia. The data entry and analysis were carried out by an independent team led by a statistician. Approval from the Ministry of Higher Education in Malaysia (Ref No. KPT.R.620-1/1/1 JId.15(9)) as well as the vice chancellors of the selected public universities and the Medical Research Ethics Committee of the Faculty of Medicine and Health Sciences, UPM (Ref No. UPM/FPSK/PADS/T7-MJKEtikaPer/F01(JKK_NOV(09)12) were obtained before the commencement of the study. A written consent was taken from each respondent before conducting the study.

### Recruitment and randomization

Malaysian female students aged 20 years old and above were recqruited. The purpose of study, date and places of screening were sent via email to the eligible students. Those who were pregnant, breastfeeding, in the final semester of their study, and/or students from the Medicine or Health Faculties were excluded.

**Fig. 2:**
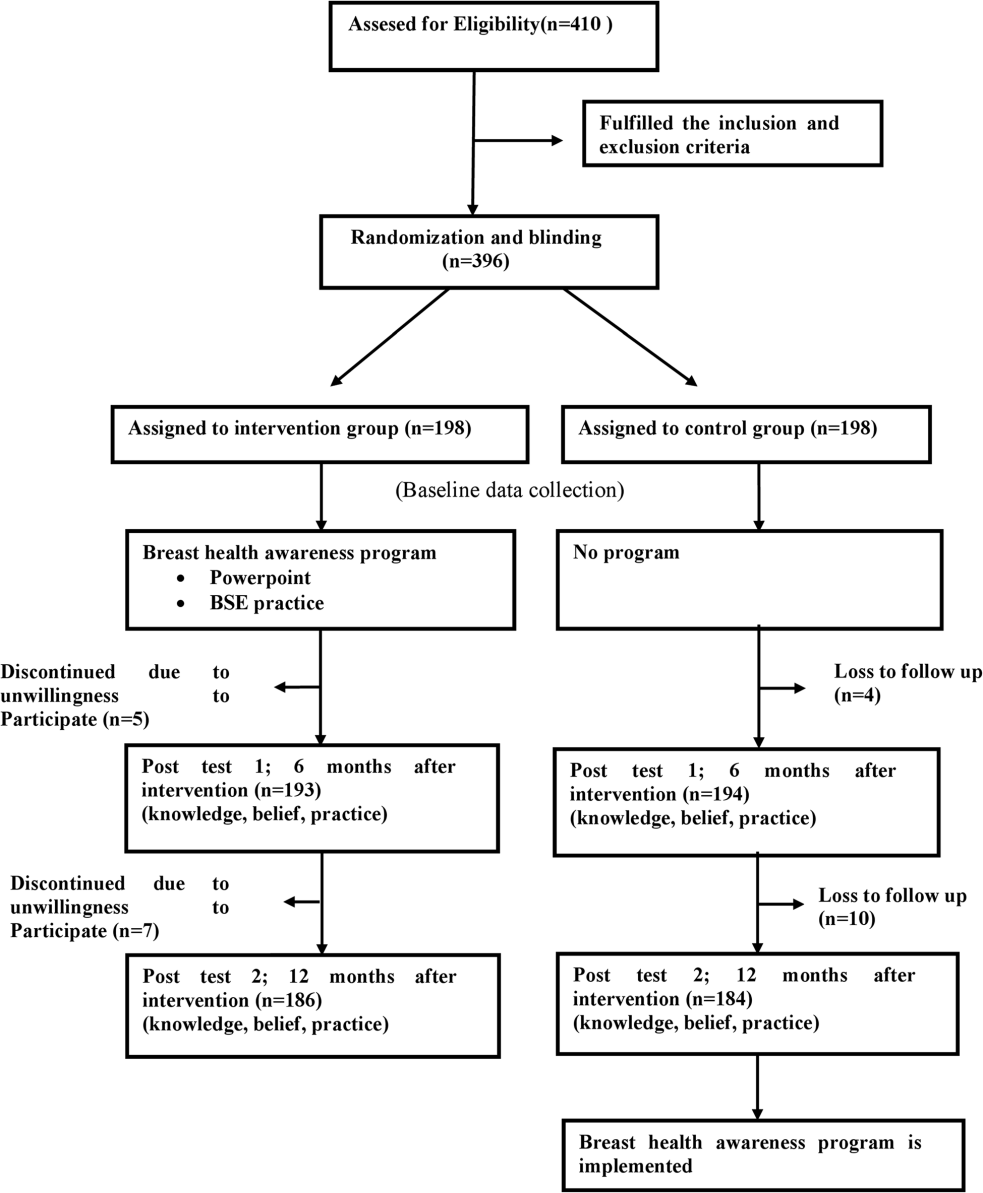
Flow chart of study participants in control and intervention groups

The list of all female undergraduate students in Department of Communication and Malay Language as well as Physical Education from the two public universities in the Klang Valley, Malaysia, served as the sampling frame. The eligible participants were assigned randomly into control and intervention groups from the sampling frame by using the random number table. Matriculation numbers were used to identify the participants in our sampling frame and unique code numbers were given to each participant in both groups and used by them in the questionnaire in order to maintain confidentiality. Figure 2 shows the flow diagram of the study participants in the control and intervention groups.

### Development of intervention

The educational module on breast health awareness was developed based on the Clinical Guidelines of Malaysia for breast cancer screening and American Cancer Society [2, 28]. The content of the education module includes the normal breast, breast health awareness, breast cancer, and other screening methods. In addition to this information, participants were trained on how to practice BSE on a silicon breast model with multiple implanted lumps.

The module was developed based on the objective of this study and peer reviewed through a series of meetings with members of the project team. The final content of the educational module and steps of the BSE practice on a silicon breast model were tested among the 30 female students other than the actual study participants for acceptability and comprehension. Table 1 summarises the topics covered in the educational module on breast health awareness.

**Table 1:** Summary of content in educational module on breast health awareness

Unit	Content
Overview	
• Introduction to content of booklet • The overall learning objectives • Target Population	
Unit 1	Breast health awareness
	• Knowledge of breast cancer • Breast self examination (BSE)
Unit 2	Normal breast
	• Anatomy of breast • Physiology of breast
Unit 3	Breast cancer
	• Symptoms of breast cancer • Risk Factors for Breast Cancer • Treatment of breast cancer
Unit 4	Other screening methods
	• Clinical Breast-Examination (CBE) • Mammography
GLOSSARY OF TERMS	
REFERENCES	

### Intervention

To enhance the participation rate in the intervention group, 16, 2-h workshops were offered, as well as a brief description of the educational module with group of 12–13 students in each.

The intervention group participated in a one-hour lecture which covered all contents of the “educational module on breast health awareness” in the form of slide presentation. Also, the intervention group received another one-hour training on breast silicon model to learn how to do the BSE practice. After the training, each participant was asked to duplicate the BSE practice on the breast silicon model to ensure they can do it correctly. At the end of the workshop, each participant was given a copy of the educational module.

The control group participants did not receive any education during the study period but the usual treatment from the health centres of each university or any campaign for breast cancer provided by the Ministry of Health Malaysia. However, they received the educational module and a training of the BSE practice on a silicon breast model after the data collection. All participants in the intervention and control groups responded to a validated and pretested questionnaire at baseline, as well as 6 and 12 months after the intervention.

### Outcome measure

The primary outcome of this study was the BSE practice. The secondary outcomes were knowledge of BSE and breast cancer as well as the health belief model scales. To evaluate these outcomes, data were collected via a self-administrative dual-language (English and Malay) questionnaire which was developed by the researchers based on the previous research publications [20, 25, 29]. The content validity was evaluated by five experts from the Community Health Department at Universiti Putra Malaysia to examine each item for congruence by estimating the Content Validity Index (CVI) as being over 0.80 (acceptable), while face validity was verified by discussing the items individually with 10 students. The reliability of the questionnaire was determined by using the test-retest reliability conducted among 80 female undergraduate students at Universiti Putra Malaysia that were not included in the main study. Data were collected using the following questionnaires:

1. *Socio-demographic data form:* Socio-demographic data consisted of age, race, religious, marital status, and family monthly income.
2. *Knowledge data form:* Participants’ breast cancer knowledge was assessed using 35 items concerning their knowledge of BSE (10 items) and breast cancer (25 items). The 25 items on the knowledge of breast cancer included general facts of breast cancer (5 items), knowledge of symptoms of breast cancer (6 items), risk factors (10 items), as well as CBE and mammography (4 items). The items were derived from the literature (25, 29, 20). Responses were measured using the nominal scale of “True”, “False” and “I do not know”. Respondents were given one point for each correct answer and zero for each wrong or unsure response. For the current study, the kappa value for categorical data was ranged between general facts of breast cancer (0.70–0.80), risk factors in breast cancer (0.52–0.97), symptom of breast cancer (0.70–0.97), CBE and mammography (0.80–0.90) and knowledge of BSE (0.70–0.87).
3. *Champion’s Health Belief Model Scale:* The third part evaluated health beliefs of the participants by using the Champion’s Health Belief Model Scale [30]. It consists of 40 questions related to the seriousness and susceptibility of breast cancer, barrier-BSE, benefit-BSE, the confidence of doing BSE and health motivation using a five-point Likert scale ranging from “strongly disagree” (1) to “strongly agree” (5) responses. Acceptable intra-class correlation coefficient (ICC) values were recorded for seriousness (0.89–0.96), susceptibility (0.79–0.86), benefit (0.85–0.98), barrier (0.70–0.80), confidence (0.88–0.97) and motivation (0.92–0.98). These values were consistent with the previous studies in Malaysia [31] and Turkish [32].
4. *BSE practice and frequency:* The last part of the questionnaire assessed the BSE practice by self-reported responses to two questions which included whether they had ever carried out BSE (yes/no) and the frequency of doing BSE (“once a month”, “occasionally”, “others” and “never”). A woman who performed BSE once a month was categorized as practicing “regular BSE” while a woman who performed occasionally or others was categorized as practicing “irregular BSE”. The Kappa value for the current study ranged from 0.82–0.85 [BSE practice (K = 0.82) and frequency of BSE (K = 0.85)].

### Sample size estimation

The sample size of this study was estimated using the Rosner’ formula (n = [zα√pq(1 + 1/k) + zβ √p1q1 + p2q2/k]2/Δ2) [33]. In order to achieve 80 % power to detect a group difference of 13 % [34] with a two-sided 5 % significance level, 165 female students in each arm were required. On the basis of a predicted attrition rate of 20 %, the goal was to randomly assign 198 female students in each intervention and control groups. Of those who initially agreed to participate in the study, 26 dropped out for variety of reasons (e.g. medical illness, unwillingness to participate, moving and schedule conflict). As a result, 186 female students in the intervention group and 184 female students in the control group completed the study in 12 months (Fig. 2).

### Statistical analysis

The data were analyzed using the Statistical Package for Social Science (SPSS) version 22.0. The outcome of interest was the BSE practice, and knowledge of breast cancer, BSE and health beliefs. The Socio-demographic characteristics of the intervention and control groups were described by using frequency, percentage, mean and standard deviation. The comparison at baseline between the intervention and control groups was made by using the appropriate inferential tests such as the Chi-square and independent samples t-test. The two-way repeated measures ANOVA (GLM) was used to evaluate the changes in the mean score of breast cancer and BSE knowledge and belief between the control and intervention groups at baseline, as well as 6 and 12 months after the intervention. The cut-off level for alpha was set at 0.05.

## Results

### Baseline data

The female students (n = 370) who participated in this study were assined to the intervention (n = 186) and control (n = 184) groups. The majority of the participants (349, 94.3 %) were Malays, whereas 21 (5.7 %) were non-Malays. The proportion of Muslims was higher than non-Muslims, 352 (95.1 %) vs. 18 (4.9 %). With regards to marital status, 357 (96.5 %) of the participants were single and 13 (3.5 %) were married. The average age of the respondents was 22 years (mean = 21.79 ± 1.24; 95 % CI = 21.66, 21.91) and the average monthly income was about RM5300 (mean = 4000 ± 2129.63; 95 % CI = 4511.74- 4947.17). At baseline, no significant difference was found between the study groups regarding participant characteristics (*p* < 0.05) (Table 2).

**Table 2:** Sociodemographic characteristics of the respondents

Characteristics	Total participants	Intervention group	Control group	Statistics
	n (%)	n (%) 186	n (%) 184	
Age				
(Mean ± SD)	21.79 ± 1.24	21.81 ± 1.38	21.76 ± 1.09	t = 0.39
				p = 0.69
Race				
Malay	349 (94.3)	174 (93.5)	175 (95.1)	χ^2^ = 0.42, p = 0.51
Non-malay	21 (5.7)	12 (6.5)	9 (4.9)	
Religious				
Muslim	352 (95.1)	174 (93.5)	178 (96.7)	χ^2^ = 2.03, p = 0.15
Non-muslim	18 (4.9)	12 (6.5)	6 (3.3)	
Marital Status				
Married	13 (3.5)	7 (3.8)	6 (3.3)	χ^2^ = 0.06, p = 0.79
Single	357 (96.5)	179 (96.2)	178 (96.7)	
Family Monthly Income				
(Mean ± SD)	4000 ± 2129.63	4540.86 ± 2013.23	4920.10 ± 2230.45	t = -1.71
				p = 0.08

*SD* standard deviation

### Change in the BSE practice and frequency

Table 3 shows the BSE practice and frequency among all participants at baseline, and 6 and 12 months after the intervention. Based on the results, 44 (23.7 %) of the participants in the intervention group practiced BSE whereas in the control group 31 (16.8 %) practiced BSE at the baseline. The rate of those who practiced BSE regularly was 15 (8.1 %) in the intervention group and 5 (2.7 %) in the control group. At baseline, no significant differences in the BSE practices (*p* = 0.10) and BSE frequency (*p* = 0.06) were found between the intervention and control group. At 6 and 12 months after the intervention, the two groups differred significantly in terms of their *BSE practice and frequency.*

**Table 3:** Changes in BSE practice and BSE frequency between intervention and control group at baseline, 6-months and 12-months after intervention

Variable	Intervention group	Control group	Statistics
	n = 186	n = 184	
	n (%)	n (%)	
BSE practice (Baseline)			
Yes	44 (23.7)	31 (16.8)	χ^2^ = 2.65, p = 0.06
No	142 (76.3)	153 (83.2)	df = 1
BSE practice (6 months)			
Yes	66 (35.5)	36 (19.6)	χ^2^ = 11.73, p = 0.001*
No	120 (64.5)	148 (80.4)	df = 1
BSE practice (12 months)			
Yes	65 (34.9)	30 (16.3)	χ^2^ = 16.84, p = 0.0001*
No	121 (65.1)	154 (83.7)	df = 1
Variable	Intervention group	Control group	Statistics
BSE Frequency (Baseline)			
Regular	15 (8.1)	5 (2.7)	χ^2^ = 5.56, p = 0.62
Irregular	29 (15.6)	26 (14.2)	
None	142 (76.3)	153 (83.2)	df = 2
BSE Frequency (6 months)			
Regular	29 (15.6)	9 (4.9)	χ^2^ = 15.00, p = 0.001*
Irregular	37 (19.9)	27 (14.6)	
None	120 (64.5)	148 (80.4)	df = 2
BSE Frequency (12 months)			
Regular	31 (16.7)	5 (2.7)	χ^2^ = 24.10, p = 0.000*
Irregular	34 (18.3)	25 (13.6)	
None	121 (65.1)	154 (83.7)	df = 2

*BSE* Breast self-examination

*Significant at *p* < 0.05

Table 4 shows changes in the BSE practice and frequency among those who never practiced BSE at baseline between groups over the period of study. Based on the results, among those who never practiced BSE at baseline, 22 (15.5 %) in the intervention group and 10 (6.5 %) in the control group practiced BSE at 6 months, while 21 (14.8 %) in the intervention group and 11 (7.2 %) in the control group practiced BSE 12 months after the intervention. Likewise, 15 (10.6 %) in the intervention group, and 2 (1.3 %) in the control group performed regular BSE 12 months after the intervention. The control and intervention groups differred significantly in the BSE practice at 6 and 12-month follow-ups (*p* < 0.05).

**Table 4:** Changes in BSE practice and BSE frequency between intervention and control group at 6-months and 12-months after intervention among those who never practice BSE at baseline

Variable	Intervention group	Control group	Statistics
	n (%)	n (%)	
BSE practice (6 months)			
Yes	22 (15.5)	10 (6.5)	χ^2^ = 6.11, p = 0.01^*^
No	120 (84.5)	143 (93.5)	df = 1
BSE practice (12 months)			
Yes	21 (14.8)	11 (7.2)	χ^2^ = 4.39, p = 0.03^*^
No	121 (85.2)	142 (92.8)	df = 1
Variable	Intervention group	Control group	Statistics
BSE Frequency (6 months)			
Regular	13 (9.2)	5 (3.3)	χ^2^ = 6.30, p = 0.04^*^
Irregular	9 (6.3)	5 (3.3)	
None	120 (84.5)	143 (93.5)	df = 2
BSE Frequency (12 months)			
Regular	15 (10.6)	2 (1.3)	χ^2^ = 11.82, p = 0.003^*^
Irregular	6 (4.2)	9 (5.9)	
None	121 (85.2)	142 (92.8)	df = 2

*BSE* Breast self-examination

^*^Significant at *p* < 0.05

### Change in the knowledge of breast cancer and self-examination

Table 5 compares the mean scores for the knowledge of breast cancer and self-examination between the intervention and control groups at baseline, 6 months and 12 months after the intervention. At baseline, there were no significant differences between the knowledge score of breast cancer (*p* = 0.66) and knowledge of breast self-examination (*p* = 0.69) between the intervention and control groups. However, the knowledge score of breast cancer and self-examination for the intervention group was significantly higher compared to the control group with mean differences of 0.83 (95%CI 0.27–1.38; *p* = 0.003) and 0.67 (95%CI 0.29–1.05;p = 0.001), respectively. Also, repeated measures ANOVA results revealed that the intervention group had statistically significant increase in the knowledge of breast cancer (F = 5.24; *p* = 0.005) and knowledge of BSE (F = 13.64;p = 0.000) over time.

**Table 5:** Mean scores of knowledge of breast cancer and knowledge of breast self-examination between intervention and control group at baseline, 6-months and 12-months after intervention

Outcome	Baseline	6-months	12-months	Eefect of intervention	*p*-value
	Mean ± SD			Mean differences (95 % CI)	
Knowledge of BC					
Intervention Group	11.32 (2.89)	12.38 (3.29)	12.41 (2.74)	0.83, (0.27–1.38)	0.003^*^
Control Group	11.53 (3.17)	11.41 (3.71)	10.69 (2.98)	0.0	
Knowledge BSE					
Intervention Group	6.29 (2.16)	6.86 (2.58)	7.79 (2.18)	0.67, (0.29–1.05)	0.001^*^
Control Group	6.39 (2.25)	6.29 (2.37)	6.23 (2.56)	0.0	

*SD* standard deviation, *CI* confidence interval

^*^Significant at *p* < 0.05

### Changes in champion health belief model scales

Table 6 presents the changes in mean scores of champion health belief model scales between the intervention and control groups. Based on the result, we found that in the intervention group, significant changes were seen from the baseline to 6 and 12 months after the intervention in the benefits of BSE (mean differences:1.09; 95 % CI 0.32–1.89; *p* = 0.006), barriers of BSE (mean differences: 0.95; 95 % CI -1.74 – -0.15; p = 0.019), confidence of BSE (mean differences: 1.66; 95%CI 0.55–2.77; *p* = 0.003) and total health belief model score (mean differences: 2.62; 95 % CI 0.03–5.21; *p* = 0.04) compared to the control group. No significant differences were found between the intervention and control groups for the rest of the components. In addition, there were no baseline differences in any component of the health belief model between the two groups.

**Table 6:** Mean scores of health belief model scales between intervention and control group at baseline, 6-months and 12 months after intervention

Outcome	Baseline	6-months	12-months	Eefect of intervention	*p*-value
	Mean ± SD			Mean differences (95 % CI)	
Susceptibility to BC					
Intervention Group	9.98 (3.92)	10.58 (3.62)	11.20 (3.62)	0.42 (-0.26–1.10)	0.22
Control Group	10.10 (3.84)	10.19 (3.24)	10.21 (3.76)	0.0	
Seriousness of BC					
Intervention Group	19.60 (4.93)	19.94 (4.50)	20.04 (4.73)	0.34 (-0.54–1.23)	0.44
Control Group	19.50 (4.33)	19.54 (4.31)	19.51 (4.14)	0.0	
Benefits of BSE					
Intervention Group	21.89 (4.19)	23.56 (3.41)	24.68 (3.74)	1.09 (0.32–1.89)	0.00^*^
Control Group	22.22 (4.58)	22.34 (4.34)	22.30 (4.23)	0.0	
Barriers of BSE					
Intervention Group	15.16 (4.69)	12.89 (3.85)	12.81 (3.95)	0.95 (-1.74–-0.15)	0.01^*^
Control Group	14.56 (4.13)	14.55 (4.28)	14.60 (4.20)	0.0	
Confidence					
Intervention Group	28.78 (7.27)	32.41 (5.48)	32.80 (7.55)	1.66 (-0.55–2.77)	0.00^*^
Control Group	29.65 (6.10)	29.71 (5.87)	29.64 (5.62)	0.0	
Health Motivation					
Intervention Group	26.52 (4.36)	26.98 (3.67)	27.67 (3.98)	0.40 (-0.41–1.21)	0.33
Control Group	26.62 (4.69)	26.70 (4.18)	26.65 (4.10)	0.0	
Total HBM score					
Intervention Group	121.64 (14.34)	126.15 (11.49)	128.88 (12.74)	2.62 (0.03–5.21)	0.04^*^
Control Group	122.72 (14.07)	123.07 (12.79)	123.01 (12.92)	0.0	

^*^Significant at *p* < 0.05

## Discussion

One of the important challenges and investments in the health of future generations of women is educating and informing youth about breast cancer [35]. Based on the Health Belief Model (HBM), this study assessed the effect of breast health education intervention on BSE practice, knowledge of breast cancer and BSE as well as health beliefs of female undergraduate students in the Klang Valley, Malaysia. Our results highlighted the importance of health education in increasing the level of knowledge among women about BSE and breast cancer, beliefs related to breast cancer and BSE, as well as BSE practice.

### BSE practice

The results of the study showed that teaching BSE practice increases its rate and the frequency of doing BSE among those who did not practice BSE before the health education program. This shows that the Breast Health Awareness program successfully motivated the women towards BSE practice. The results are in line with those of several earlier studies reporting that BSE training increases the frequency of BSE practice and performing of BSE [14, 36]. Secginli et al. [36] similarly reported that those learning BSE practice on breast silicon models comprising lumps displayed more frequent BSE practice than those who learned BSE practice through film or pamphlets. Therefore, the Breast Health Awareness program may be appropriate to increase both BSE practice and frequency of doing BSE for further samples with similar demographic characteristics.

### Knowledge of BSE

One of the hypotheses of this study was that there is a significant difference in the level of BSE knowledge among women in the intervention and control groups at 6 and 12 months post-intervention. Our results highlight the importance of health education in increasing women’s level of BSE knowledge. In accordance with the present study, other two studies carried out in Turkey [23] and Iran [37] showed that educational intervention had a positive impact on increasing the level of knowledge of BSE. The findings of this and previous studies demonstrate that health promotion education teaches young women to increase their knowledge and awareness on BSE, which is the first important step in breast cancer screening [23].

### Knowledge of breast cancer

After education, the breast cancer knowledge of participants in the intervention group significantly differed from that of the control group at all post-tests. This significant difference showed that educational intervention had a positive impact on increasing breast cancer knowledge among the participants. This finding is consistent with those of the previous studies in Iran [38], Egypt [39], India [40], Taiwan [41] which showed that educational intervention significantly increased awareness regarding breast cancer as well as the frequency of performing breast self-examination. Therefore, a community-oriented educational intervention which emphasizes proper techniques can bring a desirable change among women.

### HBM Model

In the literature, it was reported that health education is effective in increasing the breast cancer knowledge and BSE practice. However, it is difficult and complex to achieve behavioral change. In this study, positive beliefs about benefits of BSE and confidence of doing BSE were increased after health education while beliefs related to barriers of BSE decreased. This result shows that the health education intervention was effective in terms of increasing accurate perception, providing further support for the results of the previous literature [23, 35, 42]. However, the other components of health belief model such as perceived susceptibility to BC, seriousness of BC, and health motivation did not change over times, which is in line with studies done in Turkey [14] and Malaysia [43]. This may be because this study involved younger women, who believed that older women were more likely to get breast cancer; thus, they did not feel obliged to gain knowledge about breast cancer and BSE independently [44].

The participants’ confidence level and frequency of doing BSE practice increased over time in the current study. According to Selda Secginli [36] and Ceber [14], people with higher level of BSE knowledge have higher perceived confidence. Additionally, confidence in BSE is positively related to the frequency of BSE practice [43]. Consequently, promoting breast health awareness through educating women about breast cancer and teaching them how to practice BSE correctly is important. The health education methods which are used in this breast health awareness program, such as practice on breast silicon model and providing information about breast cancer via module may have fostered confidence of doing BSE and its benefit. The findings of this study are supported by Park [45] who reported that women’s confidence in performing proper BSE was improved by teaching breast awareness and BSE.

The significant difference in mean scores of perceived benefits of BSE after training in the intervention group agrees with the results of the previous studies [46, 47]. Perceived benefits of a behavior are indicative of the person’s understanding of benefits gained from conducting a behaviour [48]. The more people understand the benefits of a preventive behavior, the more they have that behaviour.

Another studied psychological factor is perceived barriers, which points out the person’s perception of intrinsic and extrinsic obstacles in performing a behaviour. Significant differences between groups were found in line with studies done in Turkey [46] and Iran [47].

## Strengths and limitations

The strengths of this study include the use of RCT, adequate sample size, low attrition rate, appropriate statistical tests and its generalizability to the college and university students in Malaysia. To the best of our knowledge, before this study no previous research is available on RCT among young female students in Malaysia; consequently, the result of this study can be used as the foundamental data for further study. Along with the numerous strengths, our study had some limitations. First of all, the result of this study cannot be generalized among all young women in Malaysia, because it focused on young educated women and was only done in public universities. It is suggested that future intervention studies should be extended to different parts and workplaces in Malaysia and among low-literate rather than educated women. Another limitation of this study is that all collected data were self-reported with no objective measures to evaluate the women. In this study, researchers did not implement any education program for the control group until the end of study. Nonetheless, the control group may have been exposed to other information sources, such as media, printed material and any campaign for breast cancer provided by the Ministry of Health during the study period which could not be controlled. In this study, although BSE practice was significantly improved after the intervention, the change was in a small number, indicating future studies should find the barriers of BSE practice and use others methods of intervention, like social media, which is more attractive for this particular group of participants.

## Implication for practice

Although there is no evidence that BSE lowers mortality from breast cancer, it should not be promoted to detect breast cancer tumors in women effectively. Women are at risk of harm from BSE including unnecessary breast biopsies, imaging tests and emotional duress [49].

Breast self-examination (BSE) might still be an important tool to improve breast awareness. Women are encouraged to take responsibility for their own health by examining themselves during bathing or dressing and to become familiar with their breasts at different times of the month and with age, looking and feeling for any changes from normal, and reporting any obvious changes promptly. Therefore, appropriate educational interventions are needed to encourage women to engage in regular breast awareness as well as to practice BSE [50].

## Conclusion

The current study demonstrates that the Breast Health Awareness program which comprised of health education materials and training of the BSE practice, was effective in increasing knowledge about breast cancer, BSE and BSE practice of the female students in the intervention group. Consequently, the Breast Health Awareness program may be appropriate for future samples with similar demographic characteristics to improve BSE in the low-resource area in Malaysia. It is also important to provide information and raise awareness about breast cancer and BSE practice among Malaysian females by the health care providers. In order to make Breast Self Examination a habit, education about breast self-examination should be started for girls at school age.

## Abbreviations

CBE, clinical breast examination; BSE, breast self-examination; BC, breast cancer; HBM, health belief model; RCT, randomized control trial, CVI, content validity index; ICC, intra-class correlation coefficient
